# The Spindle-Associated Microcephaly Protein, WDR62, Is Required for Neurogenesis and Development of the Hippocampus

**DOI:** 10.3389/fcell.2020.549353

**Published:** 2020-09-11

**Authors:** Belal Shohayeb, Uda Y. Ho, Halah Hassan, Michael Piper, Dominic C. H. Ng

**Affiliations:** School of Biomedical Science, Faculty of Medicine, The University of Queensland, St Lucia, QLD, Australia

**Keywords:** microcephaly, radial glia, hippocampus, neural proliferation, neural migration

## Abstract

Primary microcephaly genes (*MCPH*) are required for the embryonic expansion of the mammalian cerebral cortex. However, *MCPH* mutations may spare growth in other regions of the developing forebrain which reinforces context-dependent functions for distinct *MCPH* genes in neurodevelopment. Mutations in the *MCPH2* gene, *WD40-repeat protein 62* (*WDR62*), are causative of primary microcephaly and cortical malformations in humans. WDR62 is a spindle microtubule-associated phosphoprotein that is required for timely and oriented cell divisions. Recent studies in rodent models confirm that WDR62 loss or mutation causes thinning of the neocortex and disrupted proliferation of apical progenitors reinforcing critical requirements in the maintenance of radial glia. However, potential contributions for WDR62 in hippocampal development had not been previously defined. Using CRISPR/Cas9 gene editing, we generated mouse models with patient-derived non-synonymous missense mutations (WDR62^V66M^ and WDR62^R439H^) and a null mutation (herein referred to as WDR62^Stop^) for comparison. We find that WDR62 deletion or mutation resulted in a significant reduction in the thickness of the hippocampal ventricular zone and the area of the dentate gyrus (DG). This was associated with the mitotic arrest and depletion of radial glia and intermediate progenitors in the ammonic neuroepithelium. As a consequence, we find that the number of mitotic dentate precursors in the migratory stream and granule neurons in the DG was reduced with WDR62 mutation. These findings reveal that WDR62 is required for neurogenesis and the growth of the hippocampus during embryonic development.

## Introduction

*WD40-repeat protein 62* (WDR62) is a microtubule-associated signaling protein that is required for centrosome biogenesis and normal cell division or mitosis (Shohayeb et al., 2017). Initial studies on WDR62 identified functions as a scaffold protein that co-ordinated intracellular signaling through protein-protein interactions with mitogenic kinases such as c-Jun N-terminal kinase (JNK) (Wasserman et al., 2010; Bogoyevitch et al., 2012). More recently, the repertoire of kinases that interact with WDR62 has expanded to include mitotic kinases such as Aurora and Polo-like kinases (Shohayeb et al., 2017). Aurora A and JNK-mediated phosphorylation of WDR62 regulates cell cycle-dependent microtubule association and mitotic function (Lim et al., 2015, 2016). During human embryonic development, WDR62 functions in neural progenitor populations are particularly critical as inherited mutations on *WDR62* cause primary microcephaly (Bilguvar et al., 2010; Nicholas et al., 2010; Kodani et al., 2015).

Previous studies have identified numerous (>35) patient mutations in WDR62 that are causative of primary microcephaly (Shohayeb et al., 2017; Cherkaoui Jaouad et al., 2018; Yi et al., 2019). These mutations variously disrupt mRNA stability, splicing or result in the severe truncation *WDR62* to trigger nonsense-mediated decay, and lost expression (Xu et al., 2014; Shohayeb et al., 2017, 2019) highlighting that WDR62 expression is required for normal brain growth. Interestingly, patient-identified mutations of *WDR62* include a subset of atypical non-synonymous missense mutations that alter evolutionarily conserved amino acids and may specifically disrupt WDR62 function (Bilguvar et al., 2010; Nicholas et al., 2010; Shohayeb et al., 2019). In the developing CNS, WDR62 is enriched in the proliferating neuroepithelium within the cortex, the hippocampus and to a lesser extent in the cortical plate where post-mitotic neurons reside (Bilguvar et al., 2010; Sgourdou et al., 2017). In murine models, the depletion of WDR62 results in the reduced proliferative capacity of radial glia in the developing neocortex that was associated with disrupted apico-basal polarity, orientated divisions, and delayed cell-cycle progression (Chen et al., 2014; Xu et al., 2014; Jayaraman et al., 2016; Lim et al., 2017; Shohayeb et al., 2019). In addition, we showed that gene-edited mice harboring patient-identified WDR62 missense mutations (WDR62^V66M^ and WDR62^R439H^) recapitulated reduced size of whole brains and reduced cortical expansion during early-mid gestation (Shohayeb et al., 2019). This was accompanied by the reduced numbers and proliferation of radial glia (Shohayeb et al., 2019). An analysis of protein and mRNA revealed normal expression in WDR62^R439H^ mice which indicated that this mutation, located within the WD40-repeat region required for protein interactions, specifically disrupts WDR62 function and sufficient to cause cortical defects (Shohayeb et al., 2019). WDR62 is also expressed in the hippocampus and MCPH2 patients have been reported with hippocampal dysmorphology (Bilguvar et al., 2010). However, the potential impact of WDR62 missense mutations in hippocampal development has not been thoroughly explored.

The hippocampus is part of the limbic system and plays an essential role in learning and the formation of long and short-term memories (Broadbent et al., 2004; Johnston and Amaral, 2004). During development, the morphogens secreted by the telencephalon regulate radial glial cell proliferation and differentiation in the ammonic neuroepithelial layer or the ammonic ventricular zone (VZ) of the hippocampus (Barry et al., 2008). As the hippocampus develops, radial glial cells maturate and differentiate into neuronal progenitors (intermediate progenitors) which then migrate to the hippocampal *cornus ammonis* (CA) region (Barry et al., 2008; Hayashi et al., 2015). Concurrently, the DG precursors arising from the dentate neuroepithelium migrate along the dentate migratory stream to form the secondary and the tertiary matrices which ultimately populate the subgranular zone in the mature DG (Hatami et al., 2018). These migrating progenitors differentiate further to post-mitotic granule neurons in the incipient DG (Iwano et al., 2012; Hayashi et al., 2015). The glial progenitors which strongly express GFAP, however, form two glial bundles including the subgranular bundle, derived from the ammonic neuroepithelium, and the fimbrial bundle, derived from the fimbrial glioepithelium (Barry et al., 2008). These glial bundles are essential for hippocampus morphogenesis as they guide the migrating progenitors to the nascent DG (Nakahira and Yuasa, 2005; Barry et al., 2008).

Given that CDK5RAP2 and CENPJ, which are interacting partners of WDR62, have been found to alter the hippocampus and DG development (Issa et al., 2012; McIntyre et al., 2012), it is of great interest to investigate the possible implications of WDR62 mutations on the hippocampus during development. Our results revealed that WDR62 depletion or missense mutations altered hippocampus development with decreased VZ thickness and DG area. At the cellular level, the radial glial cell population was reduced in all WDR62 mutations which were associated with a decline in the developing pyramidal neurons (Tbr1^+ve^ cells) in the hippocampus CA region and the granule neurons (Prox1^+ve^ cells) in the DG. These findings demonstrate the crucial role of WDR62 in maintaining radial glia during hippocampal development.

## Results

### WDR62 Deletion or Mutation Impairs Hippocampal Development

**FIGURE 1:**
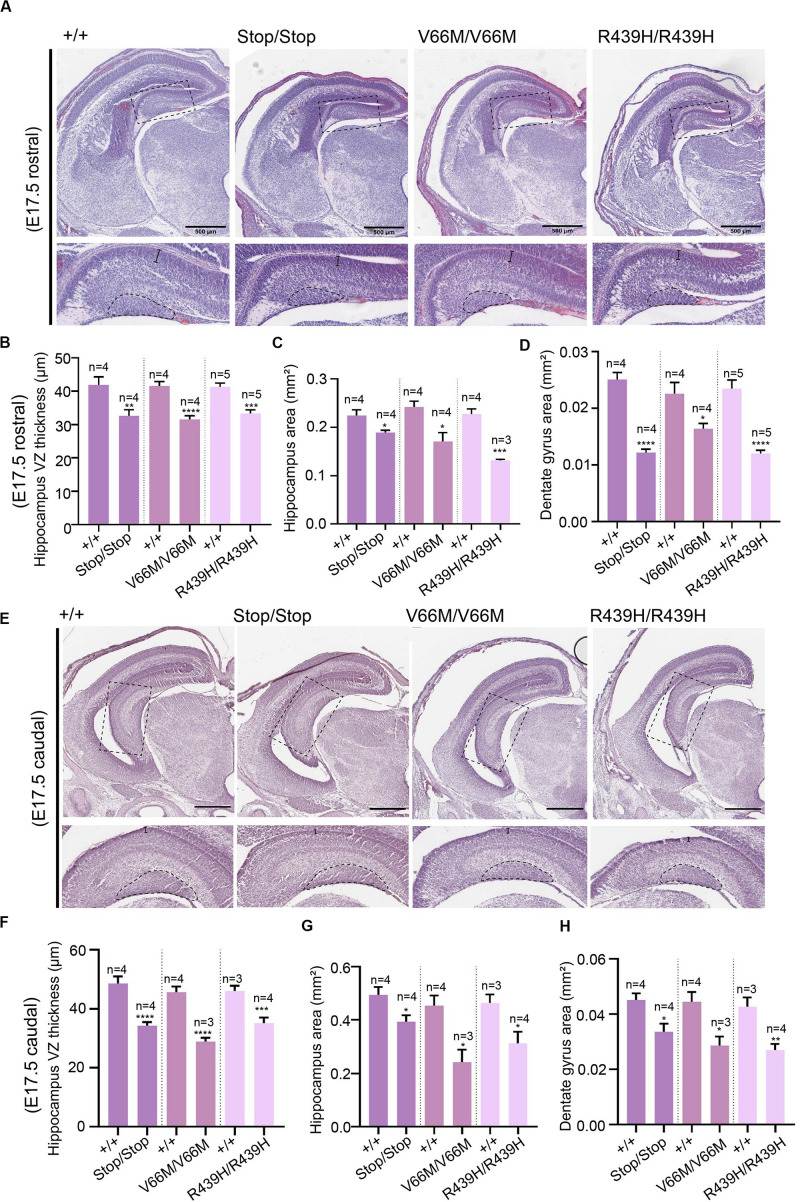
WDR62 regulates the formation of the hippocampus. **(A)** Coronal brain sections from WDR62^+/+^, WDR62^Stop/stop^, WDR62^V66M/V66M^, and WDR62^R439H/R439H^ embryos at E17.5 were stained with hematoxylin. Sections containing the rostral midline telencephalon are depicted. Hippocampal regions are shown in zoomed-in images (dash boxes) and regions for measuring VZ thickness and DG area indicated. **(B)** Quantification of VZ thickness, **(C)** area of the hippocampus, and **(D)** area of the dentate gyrus (DG). **(E)** Sections containing the caudal midline telencephalon from WDR62^+/+^, WDR62^Stop/stop^, WDR62^V66M/V66M^, and WDR62^R439H/R439H^ embryos at E17.5 are depicted. Hippocampal regions are shown in zoomed-in images (dash boxes) and regions for measuring VZ thickness and DG area indicated. **(F)** Quantification of VZ thickness, **(G)** area of the hippocampus, and **(H)** area of the dentate gyrus (DG) from caudal sections. Scale bars represent 500 μm.

**FIGURE 2:**
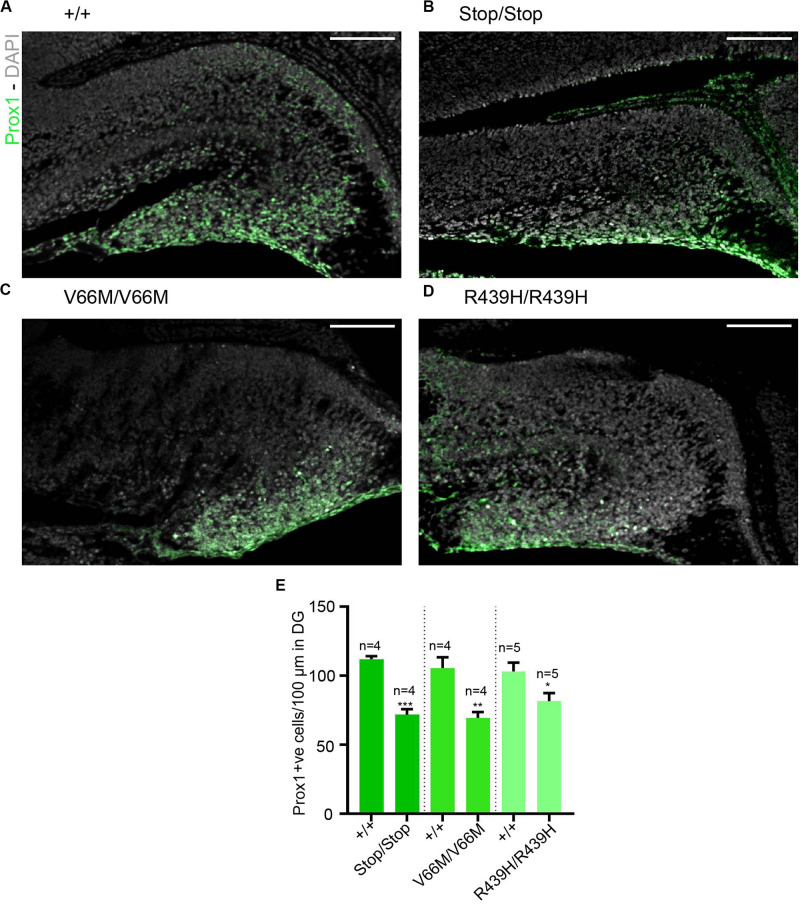
Reductions in granule neurons in the dentate gyrus with WDR62 mutations. **(A–D)** Coronal brain sections from WDR62^+/+^, WDR62^Stop/stop^, WDR62^V66M/V66M^ and WDR62^R439H/R439H^ embryos at E17.5 showing the hippocampus were stained with Prox1 (green) and DAPI (gray). **(E)** Quantification of the number of granule neurons (Prox1^+ve^) within 100 mm lineal surface of dentate neuroepithelium. Scale bars represent 100 μm.

Here, we investigate the effect of mutant WDR62, harboring patient-identified missense mutations, on the hippocampus during murine embryonic development. Using CRISPR/Cas9 gene editing, we had previously generated mouse models with pathogenic missense mutations, WDR62^V66M^ and WDR62^R439H^ or introduced a premature stop codon (WDR62^Stop^) to disrupt WDR62 expression for comparison (Shohayeb et al., 2019). Structural analysis of hematoxylin stained coronal brain sections at E17.5 revealed that the overall organization of the hippocampus and the hippocampal fissures were not grossly altered by WDR62 loss or mutation. This is in contrast to the structural defects reported in clinical cases of *MCPH2* (Bilguvar et al., 2010). However, the hippocampi were reduced in size with WDR62 loss or point mutations when compared to the wild type littermates (Figure 1A). The area of the hippocampus and thickness of the hippocampal VZ within rostral regions of the midbrain was significantly reduced in all WDR62 mutants suggesting that the numbers of radial glia (neural stem cells) that populate this region were likely reduced (Figures 1A–C). In addition, all WDR62 mutations resulted in a decrease in the size of the emerging DG indicating a reduction in the granule neurons that reside in this region (Figures 1A,D). We observed similar reductions in hippocampal VZ thickness and DG area in caudal regions of the midbrain from WDR62 mutant mice at E17.5 compared to wild-type littermates (Figures 1E–H). Consistent with a reduction in DG size, we stained for Prox1 which marks dentate granule neurons and observed a decrease in the number of Prox1^+ve^ cells with WDR62 deletion or mutations (Figure 2). We next investigated the hippocampal neuroepithelium in WDR62 mutant mice at E15.5 and found significant reductions in VZ thickness and area at this earlier developmental stage (Supplementary Figures 1A–C). Whilst hippocampal growth was impaired, the ratio of hippocampus to overall brain size was not markedly altered in WDR62 mutant mice at E15.5 and E17.5 (Supplementary Figures 1D,E,H) which indicates that reductions in hippocampal growth was not disproportionate from an overall decrease in brain size. Similarly, the ratio of DG to hippocampus area or CA region thickness relative to hippocampal VZ in rostral (Supplementary Figures 1F,G) and caudal (Supplementary Figures 1I,J) regions of the hippocampus was not significantly altered by WDR62 mutations. These results suggest that WDR62 mutations/depletion result in a deficiency in the growth of the hippocampus and the DG that is in proportion with overall reductions in brain size.

### WDR62 Mutation Perturbs Hippocampal Neural Stem Cell Populations

**FIGURE 3:**
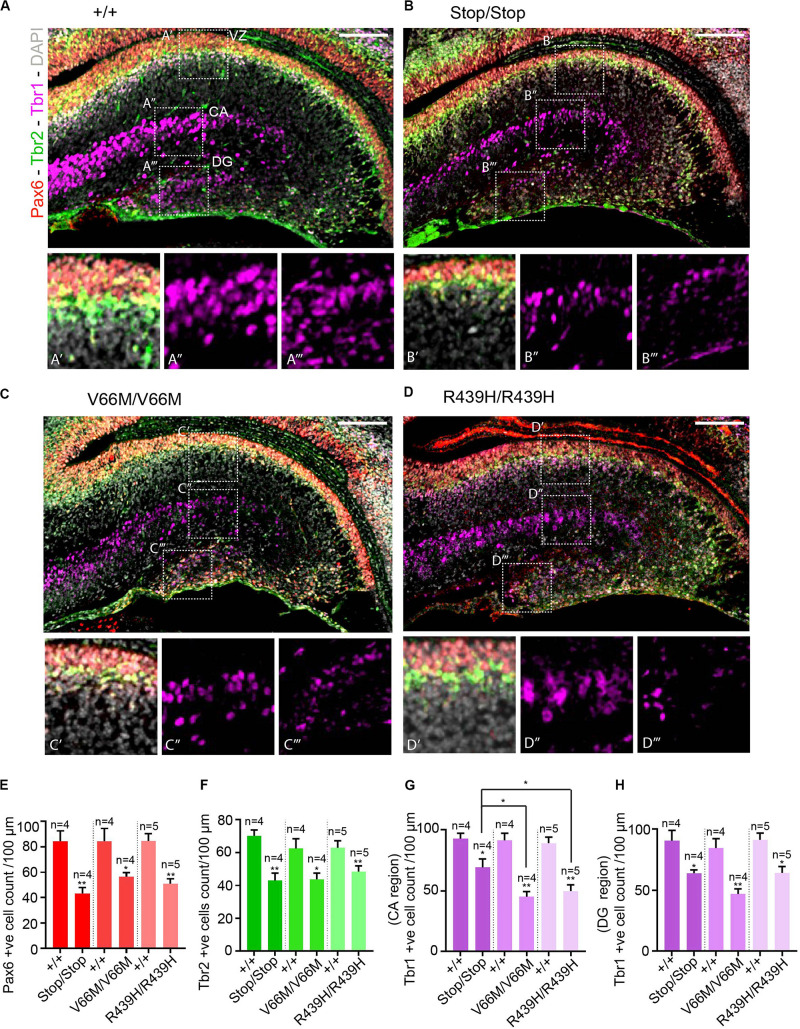
WDR62 mutation decreases radial glia and progenitor pools in the hippocampus VZ. **(A–D)** Coronal brain sections from WDR62^+/+^, WDR62^Stop/stop^, WDR62^V66M/V66M^ and WDR62^R439H/R439H^ embryos at E17.5 were stained with Pax6 (red), Tbr2 (green), Tbr1 (magenta), and DAPI (gray). **(A’–D’)** White dashed boxes more closely depict Pax6 and Tbr2 + ve cells in hippocampus VZ. **(A”–D”)** White dashed boxes more closely depict Tbr1^+ve^ cells in the hippocampal CA region. **(A”’–D”’)** White dashed boxes more closely depict Tbr1^+ve^ cells in the DG. **(E)** Quantification of radial glial (Pax6^+ve^ Tbr2^–*ve*^) cells per 100 mm of hippocampal ventricular surface. **(F)** Quantification of intermediate progenitor (Tbr2^+ve^) cells per 100 mm of ventricular surface. **(G)** Quantification of the immature neurons (Tbr1^+ve^) per 100 mm lineal surface in the hippocampal CA region. **(H)** Quantification of the immature neurons (Tbr1^+ve^) per 100 mm lineal surface in the DG. Scale bars represent 100 μm. *p < 0.05 and **p < 0.01.

**FIGURE 4:**
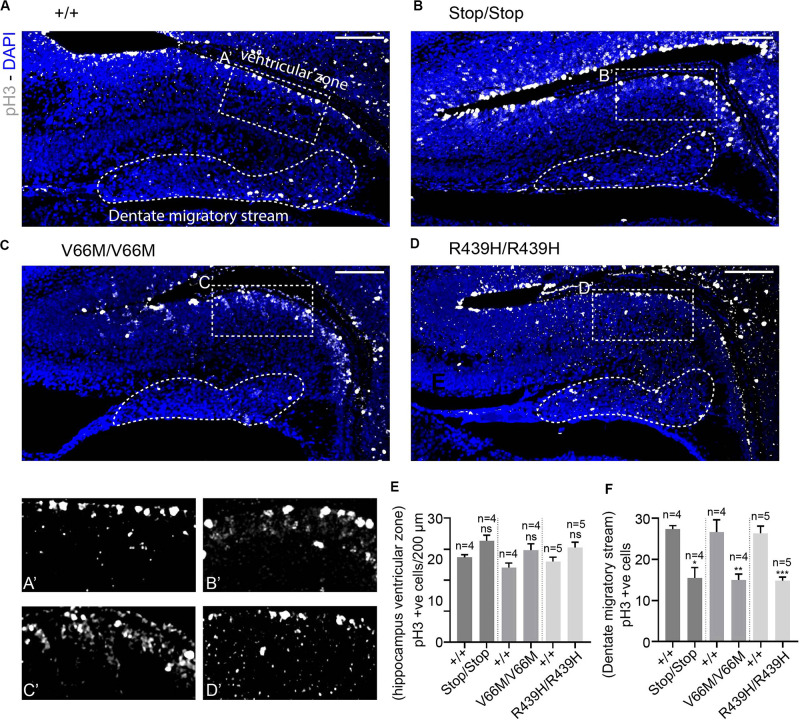
WDR62 depletion/mutations reduce mitotic cells in the dentate migratory stream. **(A–D)** Coronal brain sections from WDR62^+/+^, WDR62^Stop/stop^, WDR62^V66M/V66M^ and WDR62^R439H/R439H^ embryos at E17.5 showing the hippocampus were stained with phospho-histone H3 (pH3, Gray) and DAPI (blue) **(A’–D’)** White dashed boxes more closely depict pH3^+ve^ cells in the hippocampus VZ, The dentate migratory stream is indicated by the irregular white dashed region. **(E)** Quantification of mitotic (pH3^+ve^) cells per 200 mm hippocampal ventricular surface. **(F)** Quantification of mitotic cells in the dentate migratory stream denoted by irregular white dashed lines. Scale bars represent 100 μm. *p < 0.05, **p < 0.01, and ***p < 0.001.

To investigate the impact of WDR62 mutations on progenitor populations in the hippocampus, we stained brain sections at E17.5 for markers of radial glia (Pax6, Sox2) and intermediate progenitors (Tbr2). Our staining showed that radial glial cell numbers (Pax6^+ve^ Tbr2^–*ve*^ and Sox2^+ve^ Tbr2^–*ve*^ cells) were reduced in the hippocampus VZ of WDR62 mutant mice compared to wild-type littermates at E17.5 (Figures 3A–E,A’–D’ and Supplementary Figures 2A–E,A’–D’). We observed similar reductions in radial glia (Pax6^+ve^ Tbr2^–*ve*^) at E15.5 (Supplementary Figures 3A–E,A’–D’). This indicates that, as in the cortex (Shohayeb et al., 2019), WDR62 is involved in maintaining the radial glial cell population in the hippocampus VZ. Furthermore, corresponding with a decrease in radial glia, the number of Tbr2^+ve^ intermediate progenitors was also reduced in the hippocampus VZ in WDR62 depletion (Stop) or missense mutant (V66M and R439H) mice at E17.5 (Figures 3A–D,F) and E15.5 (Supplementary Figures 3A–D,F). Decreased numbers of intermediate progenitors appeared to be specific to the hippocampal VZ as the number of Tbr2^+ve^ cells in the dentate migratory stream was not significantly altered (Supplementary Figures 4A–E). Due to important functions in spindle regulation, the loss of WDR62 triggers mitotic arrest or mitotic delay depending on biological contexts (Ramdas Nair et al., 2016; Lim et al., 2017; Shohayeb et al., 2019). Therefore, we analyzed the number of mitotic (pH3^+ve^) radial glia in the hippocampus VZ/ammonic neuroepithelium and found a trend toward an increase in the mitotic radial glia in WDR62 mutant mice at E17.5 but this did not reach statistical significance (Figures 4A–E,A’–D’). These findings suggest that WDR62 depletion/mutation may cause a delay in the mitotic progression in neuroprogenitors residing within the hippocampus VZ resulting in their reduced numbers although this was not pronounced.

### Impact of WDR62 Mutations on the Granule Neurons

As radial glia and the intermediate progenitor numbers were reduced in the hippocampus VZ, we next investigated the population of developing pyramidal neurons in the hippocampus CA1 and CA3 regions at E17.5 through immunostaining for Tbr1, a glutamatergic neuronal marker. We found that the number of developing pyramidal neurons was decreased in the hippocampus CA region of brains from WDR62 mutant mice (Figures 3A–D,A”–D”,G). Interestingly, the reduction in Tbr1^+ve^ neurons was more severe in WDR62^V66M^ and WDR62^R439H^ mutations in comparison to WDR62^Stop^ mutation (Figure 3G) although the reason for this remains undetermined. In addition, the number of Tbr1^+ve^ neurons in the developing DG of WDR62 mutant mice was similarly reduced at E15.5 (Supplementary Figures 3A–D,A”–D”,G) and E17.5 (Figures 3A–D,A”’–D”’,H). An analysis of the cells undergoing mitosis in the dentate migratory stream indicated significant reductions in mitotic (pH3^+ve^) cells with WDR62 deletion or mutation (Figures 4A–D,F). This indicates a decrease in the production of dentate granule neurons migrating toward the DG likely due to the reduction in the neural stem cell pool in the hippocampus VZ from which these neurons arise (Nicola et al., 2015). Taken together, these studies reveal that WDR62 function is required to sustain the production and migration of granule neurons for the growth of the DG.

### WDR62 Regulates Glial Populations in the Hippocampus

**FIGURE 5:**
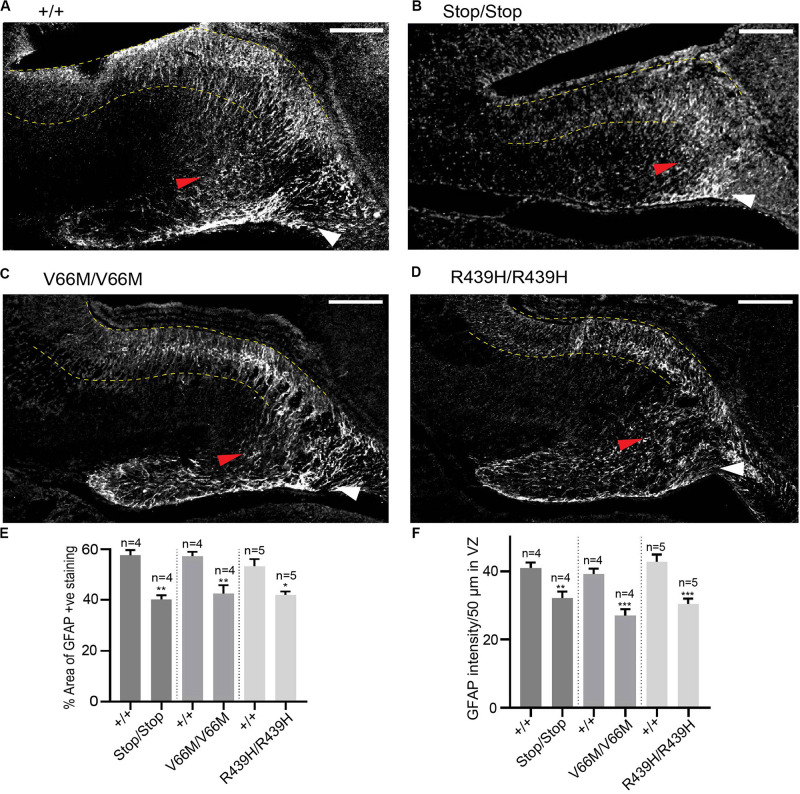
WDR62 regulate glial cell populations in the hippocampus. **(A–D)** Coronal brain sections from WDR62^+/+^, WDR62^Stop/stop^, WDR62^V66M/V66M^ and WDR62^R439H/R439H^ embryos at E17.5 were stained for GFAP (gray) to mark glia. White arrowheads indicate the glial fimbrial bundles, red arrowheads indicate the subgranular bundle and hippocampus VZ are highlighted between the 2 dashed yellow lines. **(E)** Quantification of the hippocampus positively stained with GFAP expressed as a percentage to total hippocampal area. **(F)** Quantification of GFAP intensity within a region (50 μm^2^) in the hippocampal VZ. Scale bars represent 100 μm. *p < 0.05, **p < 0.01, and ***p < 0.001.

In addition to radial glia, non-neuronal glial populations are involved in hippocampal morphogenesis as they form glial bundles along which neurons migrate to the DG and the hippocampus CA region (Barry et al., 2008). Previously, we had revealed that, in addition to neural stem cell defects, WDR62 depletion impaired the production of glial populations during *Drosophila* larval neural development (Lim et al., 2017). Therefore we next stained for GFAP, a marker for mature glia, in order to evaluate the non-neuronal glia population in the hippocampus. Both the glial subgranular bundles and the fimbrial bundle were found to exist in all WDR62 mutants (Figures 5A–D). The area of GFAP^+ve^ staining in the hippocampus, however, was significantly reduced (Figure 5E). In addition, the intensity of GFAP staining in the hippocampus VZ of WDR62 mutant mice was significantly lower when compared to wild-type animals (Figure 5F), indicating that less GFAP^+ve^ mature glia were produced from radial glia. This is likely due to the decrease in the radial glial population in WDR62 mutants as radial glia give rise to mature glia by expressing GLAST and GFAP (Barry et al., 2008). The decrease in mature glia may also contribute to the reduction of granule neurons in the DG which use glial processes as a scaffold for migration (Barry et al., 2008). Taken together, our findings indicate that WDR62 plays an essential role in maintaining the radial glia pool in the hippocampus VZ/ammonic neuroepithelium and consequently the granule neurons population in the DG and pyramidal neurons in the hippocampus CA region. Moreover, hippocampal defects observed with WDR62 deletion were recapitulated with WDR62 patient-derived missense mutations which highlight the critical loss of functions required for hippocampal neurogenesis.

## Discussion

Autosomal recessive primary microcephaly (MCPH) is clinically defined by significant reductions in brain volume and head size due to abnormal brain development (Bilguvar et al., 2010). The condition is principally attributed to pronounced deficits in the establishment and/or expansion in neural progenitors resulting in thinning of the cerebral cortex (Jayaraman et al., 2018). However, the most commonly mutated genes associated with primary microcephaly may not uniformly impact all cortical regions of the CNS. For example, a study of clinical cases with ASPM mutations reported disrupted organization and reduced volume of neocortical regions with the exception of the hippocampus and surrounding medial temporal lobe (Passemard et al., 2016). In contrast, patients harboring mutations in *WDR62*, which is second most frequently mutated in primary microcephaly, have presented with simplified hippocampal gyration and dysmorphology as part of a broad spectrum of structural malformations (Bilguvar et al., 2010; Farag et al., 2013). This suggests that, in addition to sustaining overall brain growth, MCPH genes may have region-specific functions in regulating neuroprogenitor cell populations. Previous studies have focused on WDR62 function in cortical neurogenesis (Chen et al., 2014; Xu et al., 2014; Jayaraman et al., 2016; Shohayeb et al., 2019) but WDR62 contributions to the development of the hippocampus and the DG have been extensively investigated.

*WD40-repeat protein 62* mutations result in cognitive impairment and intellectual disabilities (Nicholas et al., 2010; Yu et al., 2010; Mahmood et al., 2011), which intersect with the prognosis of some neurological disorders associated with a hippocampal impairment such as Alzheimer’s disease (Palop and Mucke, 2009). WDR62 expression in the hippocampus is observed in late embryogenesis and here we showed that CRISPR/Cas9-mediated deletion in mice (WDR62^Stop^) resulted in reduced hippocampal growth which is consistent with a previous study utilizing WDR62 depleted gene-trap animals (Sgourdou et al., 2017). Similar deficits in hippocampal growth were also observed following knock-in of single amino acid substitutions on WDR62 to recapitulate patient-identified missense mutations (WDR62^V66M^ and WDR62^R439H^). Embryonic hippocampal growth was significantly curtailed in WDR62 mutant mice and this was apparent from the reduction in hippocampal VZ thickness and DG area. The reduction in hippocampal growth appeared to be in proportion with an overall reduction in embryonic brain growth as the ratio of hippocampus to brain size was not significantly altered by WDR62 mutation. Although growth was decreased, the structural organization of the hippocampus was not grossly altered in WDR62 mutant mice. Similarly, with the exception of being marginally smaller in size, the formation of the glial subgranular and fimbrial bundles, which are composed of mature GFAP^+ve^ glia and involved in proper hippocampal morphogenesis (Gasser and Hatten, 1990; Sievers et al., 1992; Barry et al., 2008) was not substantially altered by WDR62 mutation. The shrinkage in the glial subgranular bundle is likely a consequence of decreased radial glial numbers in the hippocampus ammonic neuroepithelium as radial glia ultimately mature and differentiate into GFAP^+ve^ glia to form the glial subgranular bundles (Barry et al., 2008). The reduction in radial glia also reflects the shrinkage in the hippocampus VZ thickness. This mirrors the previously identified role for WDR62 in sustaining radial glia populations in the cortex of the developing forebrain (Bogoyevitch et al., 2012; Jayaraman et al., 2016; Shohayeb et al., 2019). Thus, our findings confirm that hippocampal growth is compromised as a result of lost protein expression in WDR62^Stop^ and WDR62^V66M^ brains or with impaired WDR62 function due to WDR62^R439H^ mutation (Shohayeb et al., 2019).

During hippocampal development, radial glia cells undergo a series of mitotic divisions either symmetrically for expansion or asymmetrically to self-renew and simultaneously produce intermediate progenitors that further differentiate into post-mitotic neurons which migrate to the hippocampus CA region or the DG (Berg et al., 2018). An analysis of mitotic cells in the hippocampal ammonic neuroepithelium indicates that they spend a longer time in mitosis with WDR62 depletion or mutation. The increase in mitoses coincides with reduced numbers of radial glia and intermediate progenitors in the hippocampus VZ. This indicates that WDR62 mutations cause mitotic defects that lead to insufficient proliferation and/or loss of radial glia and ultimately a reduction in neuroprogenitor populations in the VZ. This is consistent with a role for WDR62 in the mitotic progression of neuroprogenitors in the developing neocortex (Bogoyevitch et al., 2012; Chen et al., 2014; Ramdas Nair et al., 2016; Lim et al., 2017). Interestingly, while the number of Tbr2^+ve^ progenitors in the hippocampal VZ was decreased, their numbers in migratory stream was not substantially changed. This may reflect abnormal depletion of radial glia into Tbr2^+ve^ cells within the migratory stream similar to previous findings in the cerebral cortex (Shohayeb et al., 2019). This does not appear to translate to neuron numbers which suggests that Tbr2^+ve^ progenitor are ultimately lost without generating new neurons. Mitotic arrest leading to apoptotic cell death may contribute to a decrease in mitotic radial glia and progenitor cells although we had previously shown that WDR62 missense mutations did not significantly increase cell death in the neocortex (Shohayeb et al., 2019). Further analysis of cell death in specific progenitor populations may clarify these findings. WDR62 loss or mutation results in aberrant mitotic spindle formation leading to subsequent activation of spindle assembly checkpoint and mitotic arrest (Bogoyevitch et al., 2012; Chen et al., 2014). Furthermore, WDR62 has also been shown to be required for centrosome biogenesis. Given the importance of centrosome in spindle microtubule organization (Sanchez and Feldman, 2017), defects in centrosome numbers may also lead to errors in the microtubule-chromosome attachment to trigger mitotic arrest (Nam et al., 2015). Our study reinforces important WDR62 functions in spindle regulation, cell cycle progression and oriented divisions that are involved in sustaining self-renewal and expansion of radial glia in the hippocampus VZ.

The decline in numbers of radial glia and intermediate progenitors within the hippocampus VZ, as a result of WDR62 mutation, was associated with a concomitant reduction in developing pyramidal neurons in the hippocampus CA region and the granule neurons in the DG. The reduction in neurons in the hippocampal CA region and the DG may also be due to a perturbation in migration as neurons utilize radial glial processes and glial bundles as a scaffold for directed migration (Barry et al., 2008; Hayashi et al., 2015). In all WDR62 mutant animals, we observed a decrease in radial glia and a decrease in glial bundle area which would reasonably be expected to impact neuronal migration. Moreover, the number of mitotic cells observed in the dentate migratory stream were reduced in all WDR62 mutants which suggests reduced generation of dentate granule neurons migrating toward the incipient DG (Barry et al., 2008). Therefore, the neurogenic deficits observed in the hippocampus and DG of WDR62 mutants may be due to the combined effects of reduced neuronal migration together with mitotic/proliferative deficits in neuroprogenitor populations.

A comparison of neural deficits in animals with depletion of WDR62 with mice harboring patient-derived missense mutations revealed comparable defects in the hippocampus and the DG. A notable exception was a more pronounced decrease in Tbr1^+ve^ pyramidal neurons in the hippocampus of mice with WDR62 missense mutations compared to WDR62 depletion (WDR62^Stop^). The reason for this difference is undetermined but may be related to the compensatory increase in expression of related paralogs with overlapping functions (El-Brolosy et al., 2019). Previously we had postulated that the expression of MAPKBP1, a WD40-repeat protein that is closely related to WDR62, was elevated in response to nonsense-mediated decay in WDR62^Stop^ but not WDR62 missense mutant animals (Kodani et al., 2015). MAPKBP1 shared high-sequence conservation with WDR62, is similarly expressed in the hippocampus (Allen Brain Atlas) and is localized to mitotic spindle poles (Macia et al., 2017). An analysis of MAPKBP1 expression in WDR62 mutant hippocampi may resolve differential effects on neuronal numbers observed with protein knockout versus single amino-acid substitutions. Taken together, our findings reveal the critical role of WDR62 in hippocampal growth and demonstrate the impact of patient-identified mutations on hippocampal neurogenesis during embryonic development.

## Experimental Procedures

### Generation of Mouse Lines

The WDR62^Stop^, WDR62^V66M^, and WDR62^R439H^ mutant mice were generated by the Australian Phenomics Network (Monash) through CRISPR/Cas9 editing. The following are the animal ethics approval numbers (SBMS/AIBN/445/18 and SBMS/AIBN/375/15/NHMRC/ARC) obtained from the animal ethics unit at the University of Queensland. More details about the generation of these mouse lines and genotyping can be found in our previous study (Shohayeb et al., 2019).

### Hematoxylin Staining

E17.5 brains were processed in paraffin-wax and cut at 10 μm using a microtome. Following dewaxing, brain sections were stained with Mayer’s hematoxylin, washed and fixed in xylene. Brain sections were then mounted in DePeX mounting media and imaged by Aperio slide scanner.

### Immunohistochemistry

Brain sections were dewaxed and antigens were retrieved at 95°C for 15 min in 10 μM pH 6.0 sodium citrate buffer using a decloaking chamber. Following antigen retrieval, brain sections were incubated for an hour in a blocking buffer (20% FCS + 2% BSA + 0.2% TritonX in 50 mL PBS, filtered). The primary antibody solution was applied overnight. After washing the primary antibodies, the secondary antibody solution was applied for 2 h. Primary antibodies used for immunohistochemistry were rabbit anti-Tbr1 (1:200, ab31940 Abcam), rabbit anti-Tbr2 488 (1:200, 53-4875-80 eBioscience), rabbit anti-Prox1 (1:300, ab101851 Abcam) rabbit anti-pH3 (1:300, ab47297 Abcam), rabbit anti-Pax6 (1:200, DSBH), rabbit anti-GFAP (1:200, ab7260 Abcam) and rabbit anti-Sox2 (1:200, ab196175 Abcam). The secondary antibodies used were anti-rabbit Alexa Fluor 488, anti-rabbit Alexa Fluor 555, anti-mouse Alexa Fluor 555 and anti-rabbit Alexa Fluor 647 (1:400, eBioscience). Following immunohistochemistry, each brain section was imaged as z-stacks (10 μm) using a Leica SP8 laser scanning confocal microscope using high NA 20x and 40x objectives.

### Data Quantification and Statistical Analysis

The hippocampal, DG and brain area and the thickness of the hippocampal VZ were quantified in hematoxylin stained brain sections with ImageScope. Radial glia and progenitor cells within a region of interest (typically 100–200 μm lineal surface of neuroepithelium) were counted in ImageJ. Typically, 2 regions of interest were measured per brain section and 3 sections were imaged per animal. “n” – denotes number of animals quantified. Statistical analyses were performed in GraphPad Prism using a two-tailed unpaired student’s *t*-test. All the error bars are represented in the standard error of the mean (SEM). Significance stars included in graphs are as follows ^∗^*p* < 0.05, ^∗∗^*p* < 0.01, and ^∗∗∗^*p* < 0.001.

## Data Availability Statement

The raw data supporting the conclusions of this article will be made available by the authors, without undue reservation, to any qualified researcher.

## Ethics Statement

The animal study was reviewed and approved by the University of Queensland Anatomical Biosciences Animal Ethics Committee (SBMS/AIBN/445/18 and SBMS/AIBN/375/15/NHMRC/ARC).

## Author Contributions

BS executed the experiments, analyzed the data, and wrote the manuscript. UH and HH contributed to generation of experimental data and editing of the manuscript. MP contributed to data analysis and interpretation and helped to edit the manuscript. DN conceived of experiments, contributed to data analysis and interpretation, and writing the manuscript. All authors contributed to the article and approved the submitted version.

## Conflict of Interest

The authors declare that the research was conducted in the absence of any commercial or financial relationships that could be construed as a potential conflict of interest.
