# Supplementary material for: The Spindle-Associated Microcephaly Protein, WDR62, Is Required for Neurogenesis and Development of the Hippocampus
Source: Front Cell Dev Biol. 2020 Sep 11;8:549353. doi: 10.3389/fcell.2020.549353 (PMC7517699; doi:10.3389/fcell.2020.549353)
Supplement: Supplementary file 1 [file Image_1.pdf]

Supp Figure 1

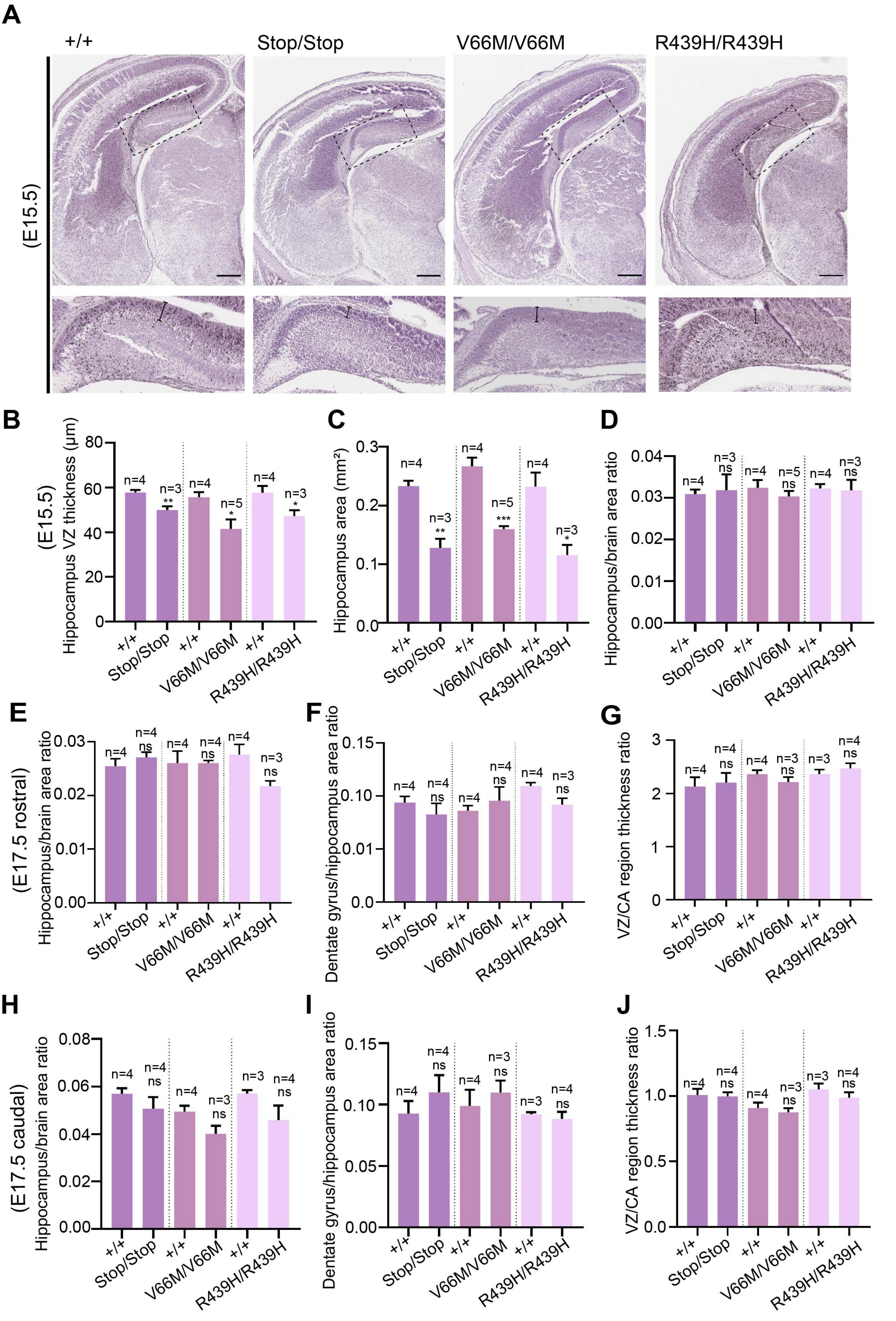

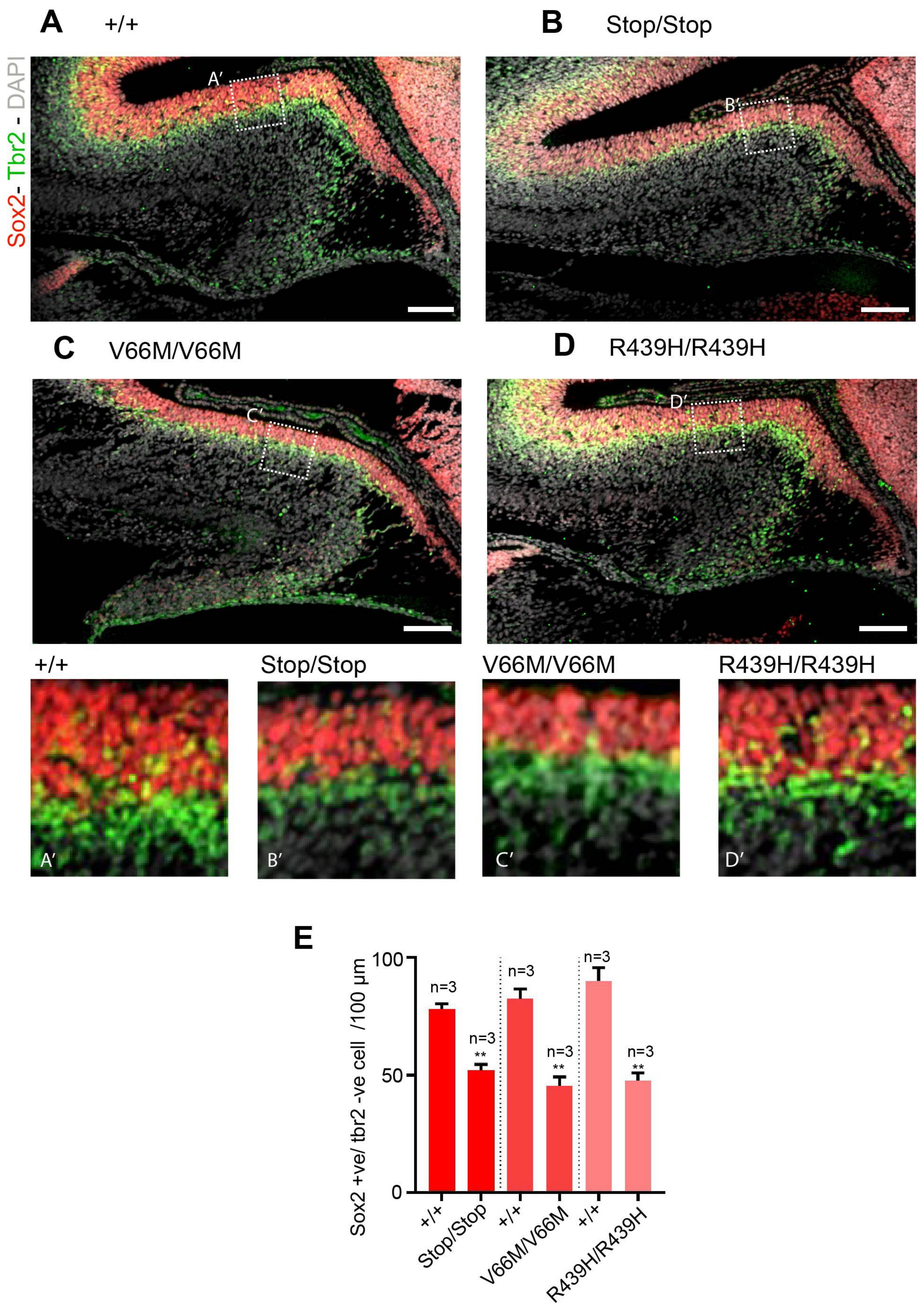
A
+/+
B
Stop/Stop
Sox2- Tbr2 - DAPI
A′
B′
C
V66M/V66M
D
R439H/R439H
C′
D′
+/+
Stop/Stop
V66M/V66M
R439H/R439H
A′
B′
C′
D′
E
Sox2 +ve/ tbr2 -ve cell /100 µm
100
50
0
n=3
n=3
**
n=3
n=3
**
n=3
n=3
**
+/+
Stop/Stop
+/+
V66M/V66M
+/+
R439H/R439H

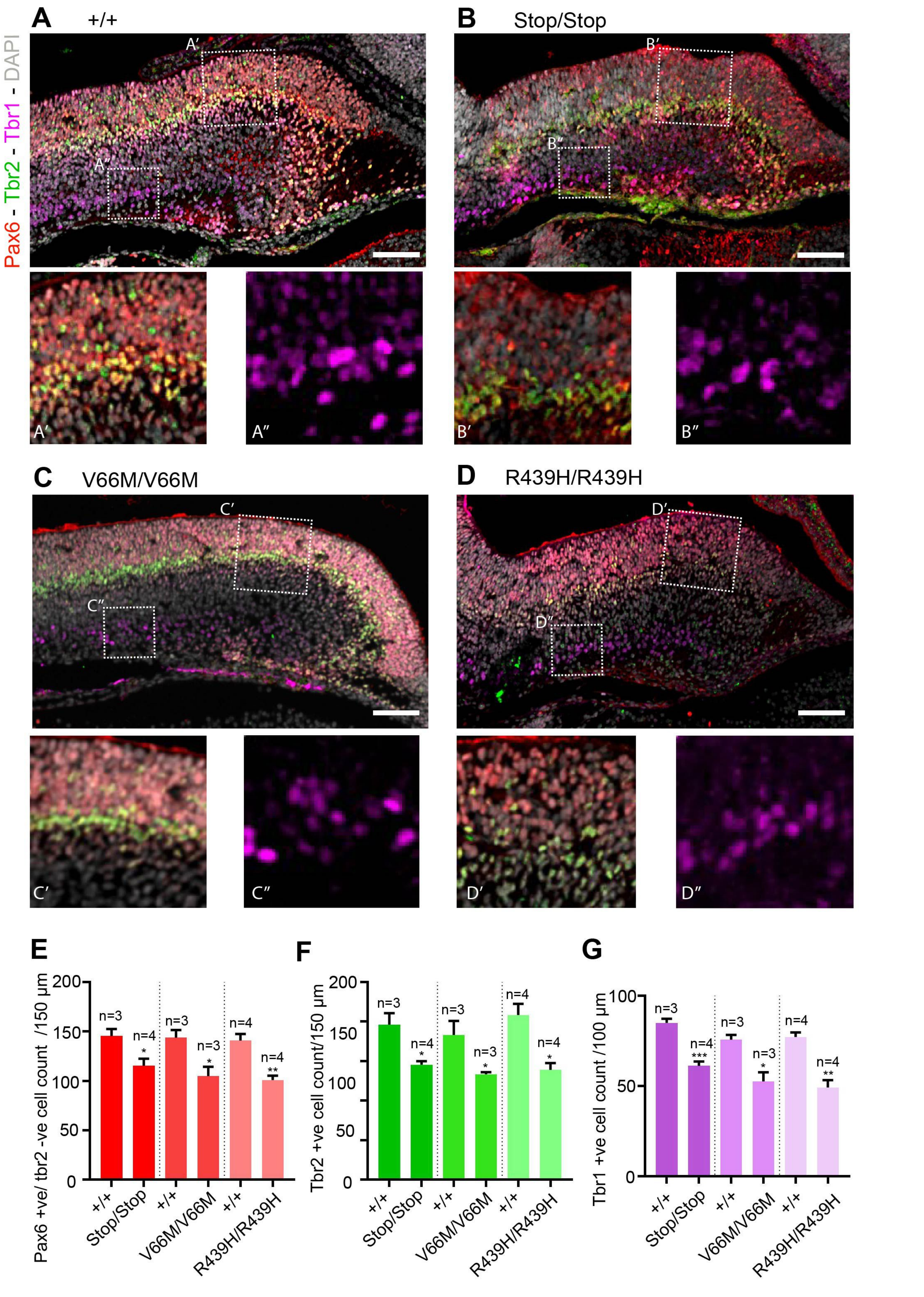
A +/+
B Stop/Stop
Pax6 - Tbr2 - Tbr1 - DAPI
A′
A″
B′
B″
C V66M/V66M
D R439H/R439H
C′
C″
D′
D″
E
F
G
Pax6 +ve/ tbr2 -ve cell count /150 µm
Tbr2 +ve cell count/150 µm
Tbr1 +ve cell count /100 µm
n=3
n=4
*
**
***
+/+
Stop/Stop
V66M/V66M
R439H/R439H
0
50
100
150
200

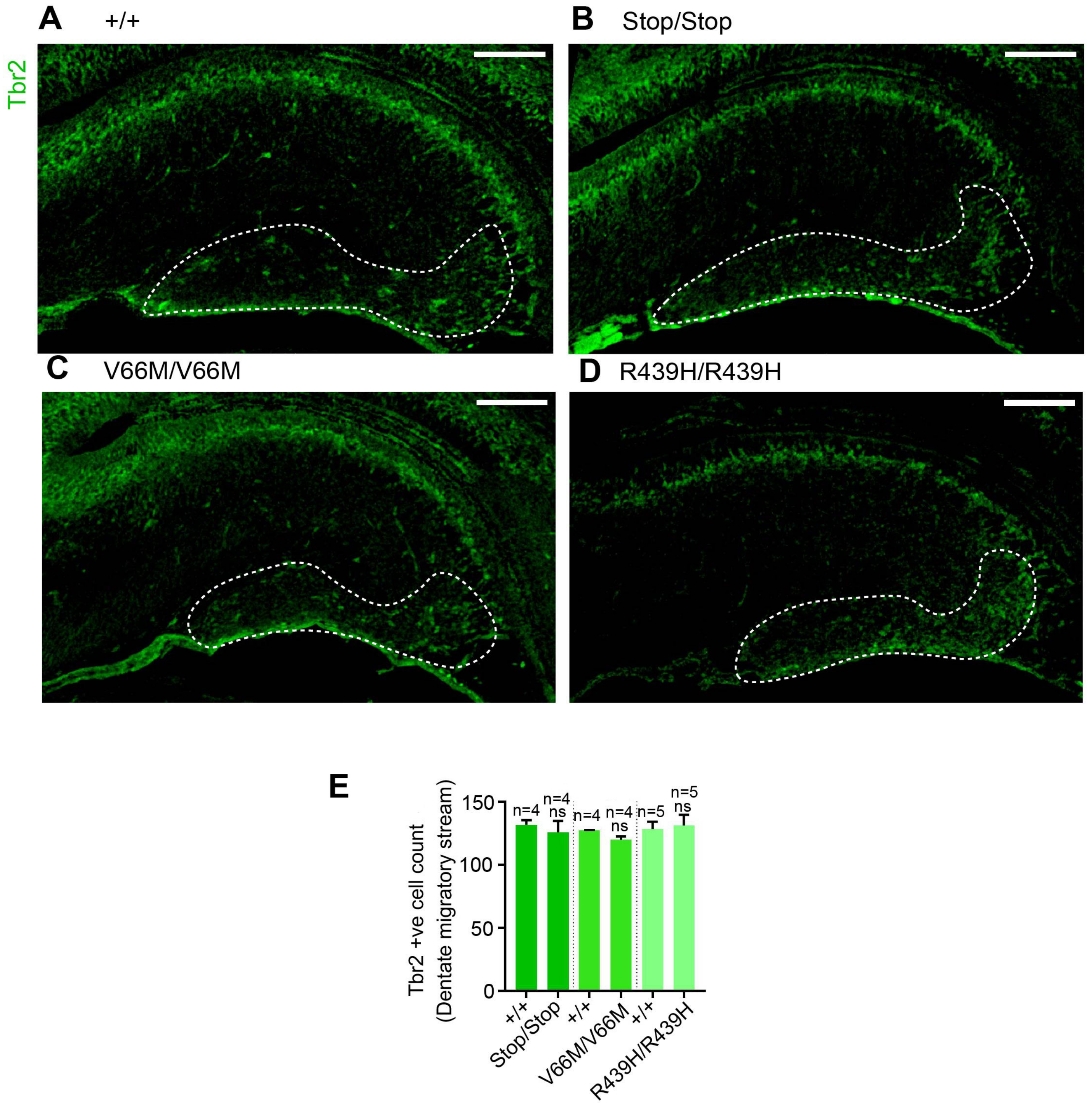
A
+/+
B
Stop/Stop
Tbr2
C
V66M/V66M
D
R439H/R439H
E
Tbr2 +ve cell count
(Dentate migratory stream)
150
100
50
0
n=4
n=4
ns
n=4
n=4
ns
n=5
n=5
ns
+/+
Stop/Stop
+/+
V66M/V66M
+/+
R439H/R439H

Supplementary Figure Legends

Supplementary Figure 1. WDR62 mutations impair hippocampal development.

Coronal brain sections from $WDR62^{+/+}$, $WDR62^{stop/stop}$, $WDR62^{V66M/V66M}$ and $WDR62^{R439H/R439H}$ embryos at E15.5 were stained with haematoxylin. Hippocampal regions are shown in zoomed-in images (dash boxes) and regions of VZ thickness measured are indicated. **(B)** Quantification of VZ thickness and **(C)** area of the hippocampus. **(D)** Hippocampal area expressed as a ratio of brain area at E15.5. **(E)** Hippocampal area expressed as a ratio of brain area, **(F)** dentate gyrus area relative to hippocampal size and **(G)** hippocampal VZ thickness relative to CA region thickness quantified from coronal sections from rostral midline telencephalon at E17.5. **(H)** Hippocampal area expressed as a ratio of brain area, **(I)** dentate gyrus area relative to hippocampal size and **(J)** hippocampal VZ thickness relative to CA thickness quantified from coronal sections from caudal midline telencephalon at E17.5.Scale bars represent 500 μm.

Supplementary Figure 2. WDR62 mutations reduce the number of $Sox2^{+ve}$ radial glial cells in the hippocampus.

(A-D) Coronal brain sections from $WDR62^{+/+}$, $WDR62^{stop/stop}$, $WDR62^{V66M/V66M}$ and $WDR62^{R439H/R439H}$ embryos at E17.5 were stained with Sox2 (red), Tbr2 (green), and DAPI (grey). **(A'-D')** White dashed boxes more closely depict Pax6 and Tbr2 stained cells in hippocampus VZ. **(E)** Quantification of $Sox2^{+ve}$ $Tbr2^{-ve}$ cells per 100 μm of hippocampal ventricular surface.

Supplementary Figure 3. Neural stem and progenitor cell deficiencies in hippocampus of WDR62 mutant embryos.

(A-D) Coronal brain sections from $WDR62^{+/+}$, $WDR62^{stop/stop}$, $WDR62^{V66M/V66M}$ and $WDR62^{R439H/R439H}$ embryos at E15.5 were stained with Pax6 (red), Tbr2 (green), Tbr1 (magenta) and DAPI (grey). **(A'-D')** White dashed boxes more closely depict Pax6 and Tbr2 stained cells in hippocampus VZ. **(A"-D")** White dashed boxes that more closely depict $Tbr1^{+ve}$ cells in the developing DG**.** **(E)** Quantification of radial glial ($Pax6^{+ve}$ $Tbr2^{-ve}$) cells per 150 μm of hippocampal ventricular surface. **(F)** Quantification of intermediate progenitor ($Tbr2^{+ve}$) cells per 150 μm of hippocampal ventricular surface. **(G)** Quantification of the immature neurons ($Tbr1^{+ve}$) per 100 μm lineal surface in the DG. Scale bars represent 100 μm.

Supplementary Figure 4. Intermediate progenitor numbers in the dentate migratory stream are not altered by WDR62 mutations.

(A-D) Coronal brain sections from WDR62$^{+/+}$, WDR62$^{stop/stop}$, WDR62$^{V66M/V66M}$ and WDR62$^{R439H/R439H}$ embryos at E17.5 were stained with Tbr2 (green). The dentate migratory stream is indicated by irregular white dashed region. **(E)** Quantification of intermediate progenitor (Tbr2^{+ve}) cell numbers in the dentate migratory stream.
